# Historical Context of Cardiac Rehabilitation: Learning From the Past to Move to the Future

**DOI:** 10.3389/fcvm.2022.842567

**Published:** 2022-04-27

**Authors:** Julie Redfern, Robyn Gallagher, Adrienne O’Neil, Sherry L. Grace, Adrian Bauman, Garry Jennings, David Brieger, Tom Briffa

**Affiliations:** ^1^Faculty of Medicine and Health, University of Sydney, Sydney, NSW, Australia; ^2^School of Medicine, IMPACT Institute, Deakin University, Geelong, VIC, Australia; ^3^Faculty of Health, York University, Toronto, ON, Canada; ^4^KITE Toronto Rehabilitation Institute and Peter Munk Cardiac Centre, University Health Network, University of Toronto, Toronto, ON, Canada; ^5^School of Public Health, University of Sydney, Sydney, NSW, Australia; ^6^National Heart Foundation of Australia, Melbourne, VIC, Australia; ^7^Department of Cardiology, Concord Hospital, Sydney, NSW, Australia; ^8^School of Population and Global Health, University of Western Australia, Perth, WA, Australia

**Keywords:** cardiac rehabilitation, secondary prevention, digital health, data, heart

## Abstract

Contemporary myocardial infarction (MI) care and management has evolved dramatically since the 1950’s; yet outpatient rehabilitation remains underutilized. Deepening our understanding of the origins and history of cardiac rehabilitation highlights a contemporary shift required for policy and practice related to secondary prevention of coronary disease in light of societal changes as well as medical, digital and surgical advancements. Contemporary “cardiac rehabilitation” began when bed rest and physical inactivity was recommended and commonplace for MI survivors. Today, most patients who survive an MI, undergo reperfusion therapy, a short inpatient stay and are discharged with minimal physical morbidity. Despite this, the majority of modern day programs continue to be structured in the same way they have been for the past 50 years and this model has become incongruent with the contemporary context, especially in the COVID-19 era. This review aims to describe the historical foundations of cardiac rehabilitation to inform solutions and meet the demands of contemporary MI management. Delivering health systems reform to address modernization is current healthcare challenge where a united and interdisciplinary effort is needed.

## Introduction

Cardiovascular disease (CVD), including coronary heart disease (CHD) and stroke, is the leading cause of death and disease burden globally (1). It is estimated that 32% of deaths internationally in 2019 were due to CVD (approximately 17.9 million deaths) (1). Based on analysis of epidemiological data from the Global Burden of Disease dataset, ischemic heart disease (IHD) affects around 126 million individuals globally, which is approximately 1.72% of the world’s population (2). In 2017, IHD was identified as the leading international cause of death (estimated 9 million) (2). People with a previous diagnosis of CVD are at the greatest risk of repeat events and data suggests that around one quarter will have another CVD event requiring admission to hospital within in the first year of an acute coronary event (3, 4). The good news is, over the past 75 years, there have been major developments in management in terms of how a diagnosis is made, how coronary arteries are revascularized and what medications are available to patients. These advancements have resulted in more patients surviving initial events, reduced length of stay in hospital which in turn means there are escalating numbers of people requiring ongoing and lifelong cardiovascular risk management (5). As such, international groups and organizations have identified improved secondary prevention as an international priority (6, 7).

**FIGURE 1:**
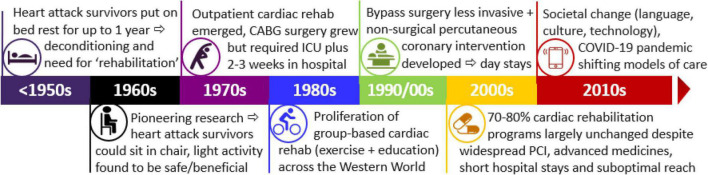
Timeline for cardiac rehabilitation in the context of acute care and transformation and societal change. PCI, percutaneous coronary intervention; CABG, coronary artery bypass graft surgery; ICU, intensive care unit.

Understanding the historical context can inform our understanding of cardiac rehabilitation and its potential in the future. This includes deepening understanding of why current programs are formatted as they are and how this has failed to adapt with changed needs of societies where there has been major changes in culture, language and diversity coupled with a rapid expansion in availability of technology along with major changes in medical and surgical management of CVD in recent years. The aim of this review is to summarize the historical context of cardiac rehabilitation in order to highlight areas for modernization and reform. That is to put in context the timing of changes in acute care and the lack of change in cardiac rehabilitation during the same time period highlighting the subsequent gaps in health services and systems at the present time. The overall timeline is summarized in Figure 1.

## Historical Approach to Myocardial Infarction: Identification to 1950s

The early descriptions of myocardial infarction (MI) and its associated treatment evolved dramatically around the mid-twentieth century. The coronary circulation was first recognized in the early 17th century and angina pectoris first described in the 18th century, but it was not until the late 19th century that further research identified the link between coronary artery occlusion and MI (8). In a landmark paper published in 1912, James Herrick was the first to claim that MI was not necessarily fatal and documented the importance of total rest as treatment (9). This early work led to a new treatment paradigm for patients who experienced MI in the first half of the 18th century that recommended “*absolute rest in bed for not less than a month is imperative to allow healing of the infarct and to reduce the risk of embolism*…*.convalescence will therefore be prolonged and the return to ordinary life postponed as long as possible*” (10). However, while a 1938 paper described the importance of bed rest for congestive heart failure it also acknowledged that understanding of the amount of bed rest required for patients was unclear (11). However, up until the mid 20th century, after MI, patients who survived were required to stay confined to bed for over 6 weeks including being prohibited from walking to the bathroom independently (12). Once discharged from hospital, severely limited physical activity was prescribed, with functional tasks (including walking up stairs) being forbidden for 12 months in some cases (13). Therefore, for the whole first half of the 20th century, management of MI focused almost exclusively on complete *physical inactivity*.

## Questioning Bed-Rest Post-MI: 1950s and 1960s

By the mid 20th century, MI had become widely understood as a common cause of death and a significant health concern (14). However, bed-rest recommendations were also known to be associated with the sequelae of immobility including muscle atrophy, functional deconditioning and fear of activity coupled with increased risk of deep venous thrombosis and pulmonary embolism (15). In the early 1950s, two American physicians (Levine and Lown) began to probe the need for prescribed bed-rest and prolonged inactivity after MI and explored the possibility of what became know as “armchair” treatment (16). This treatment allowed patients to sit in a chair for 1–2 h a day (16). The treatment was pervasive and unconventional but Levine argued it also improved “mental state”(17). Following these developments, supportive evidence progressively emerged and eventually led to a changes in post-MI management where patients were allowed to progressively increase walking and function (12). By the 1960s, several studies had reported that light activity after MI was safe and indeed beneficial in terms of preventing the negative effects of extended immobility (12). This aligned with emerging research in other areas of health where researchers were beginning to report that regular and supervised exercise programs (two times a day for 3 months) could help overcome the deleterious effects of immobility and associated deconditioning (18).

**Box 1. Quotes from an interview about the emergence of cardiac rehabilitation in the 1970s with Sister Doreen Hennesy who was “Sister-in-Charge” of a Coronary Care Unit in Sydney, Australia (26).**

“In 1972 when I became the Sister-in-Charge of the Coronary Care Unit at Parramatta, cardiac monitoring and this type of thing was very new and exciting and we so conquered the cardiac arrest situation…. So a lot more patients were living that would have died. However, they weren’t living. They were wrapped in cotton wool, they were scared, they were terrified. So I started an education program of the patient while they were in hospital, explaining what the heart attack was to them in lay terms.”

“I started early ambulation in 1975 where we were getting patients out of bed within 2 days of their admission. This was unheard of.”

“In 1978 we exercised the first patient, ten days after an ‘infa’ (infarction). It was exciting. It was everything that I ever wished to do. It was also very frightening. Although, I had seen it all working in Canada and knew it was safe, the first patient was exercised in front of doctors from the Heart Foundation, the medical directors and physicians from Parramatta Hospital and I was just there with one bike and a little machine.”

“Within two months, I had about forty patients and I was just one staff. Then it grew and then I got more staff, more patients. At some stage we had 65 patients a day, just in a session in the evening where we used run the cardiac gymnasium. It was also really a lot of fun; the nursing staff did it in their own time and we used the hospital’s equipment. Sometimes, we had up to 80 patients in an evening, just coming in skipping rope, bench stepping using some of the equipment, calisthenics; all this was done by these cardiac patients.”

## The Birth of Cardiac Rehabilitation, Alongside Acute Cardiac Treatments: 1960s/1970s

By the 1970s, a model of structured “rehabilitation” for patients with CHD was progressively introduced around the world. A new area of research and clinical practice had emerged with numerous groups commencing research investigating potential benefits and safety of the group-based approach (13). Availability of medicines and the use of oxygen during exercise also evolved (19). One controversial study at the time, published in 1968, found that MI survivors benefited from participating in an exercise program both physically and physiologically without increasing risk of death or further events (20). The benefits of supervised exercise programs provided a new approach to post-discharge care and eventually evolved into what we know today as outpatient or traditional “cardiac rehabilitation.” This concept of “rehabilitation” was a logical progression, where patients who survived a MI, required a period of supervised exercise to overcome the deconditioning associated with previously recommended treatment.

By the mid-1970s, cardiac rehabilitation programs had emerged in approximately 25 countries (21). These programs started primarily as an inpatient model but eventually progressed to outpatient programs that involved supervised physical activity sessions requiring a low-level of oxygen demand (13). As identified by Buckley, the standard approach to “rehabilitation” at this time focused on exercise with only a few some programs addressing psycho-social care (22). In Canada, early research found that men with CHD could safely participate in supervised exercise programs after MI with a small group training for and completing the Boston Marathon (23, 24). Recommendations at the time were focused on exercise and included stating that “the physician is responsible for both the safety and effectiveness of the exercise prescription” and that “all exercise should be supervised…with sessions once to twice per week for one year”(25). Box 1 highlights the feeling of Australian health professionals about the emergence of cardiac rehabilitation in the 1970s (26). At the same time a survey in Britain indicated that there were no specific cardiac rehabilitation facilities although 8% (nine hospitals) of respondents reported there was some form of exercise program but on further investigation this seemed focused on early mobilization and/or physiotherapy and exercise regimens during hospital stay (27). Overall, although 74% of respondent cardiologists were in favor of a service there was a strong focus on the need for “*individual instruction by the physician*” and the focus was on exercise although several noted the importance of “*psychotherapy*” and “*individual advice*” although funding was identified as a barrier (27).

Also in the 1970s, the Framingham Heart Study had identified risk factors for CVD and their role in prevention and management was becoming widely acknowledged (28). The Framingham study had followed a large cohort of participants over a long period and eventually identified a variety of modifiable risk factors for CVD (28). The identified risk factors included high blood pressure, high blood cholesterol, tobacco use, obesity, diabetes, and physical inactivity, psychosocial issues along with non-modifiable factors including age, gender and genetic disposition (29). Theses factors subsequently became an integral part of primary and secondary prevention of CVD (29). This focus also increased emphasis on the importance of physical activity and exercise in addressing multiple risk factors and hence the evolving rehabilitation programs initially were mostly exclusively exercise-only but over time they progressively included multidisciplinary education and psychosocial support for patients.

At this time, advances and developments were also made in terms of medications with the potential benefits of thrombolytic agents, statins and antiplatelet medicines amongst others (29, 30). Major developments were also underway in the area of coronary artery bypass surgery (CABG). CABG was clearly a breakthrough in the care of patients with coronary disease, but it was and remains an invasive surgical procedure that requires cardiopulmonary bypass during surgery, along with sternotomy, mechanical ventilation and an intensive care stay (31). These requirements of course prolonged post-operative recovery as well as advice regarding return to physical activity and function (31). Early CABG required lengthy stays in intensive care units and hospital stays of several weeks and an ongoing need for inpatient ambulation and outpatient prescriptive exercise for recovery (32). Further medical advancements saw coronary angioplasty first used in humans in Switzerland in 1977, which progressed to current routine use of percutaneous coronary intervention (PCI) that was minimally invasive with only a brief hospital stay and rapid return to normal activity and work (33). As such, “rehabilitation” needs post-PCI were (and remain) vastly different to the needs of patients who underwent CABG.

## Proliferation of Group-Based Cardiac Rehabilitation: 1980s and 1990s

By the late 20th century, group-based, outpatient cardiac rehabilitation had become commonplace in many developed countries (21). By 1980, cardiac rehabilitation was reported to be running in an estimated 30 countries and by 2000 this had increased to almost 60 countries covering all continents (21). In 1993, a World Health Organization Expert Committee on rehabilitation after CVD identified that “rehabilitation is considered to be an essential part of the care that should be available to all cardiac patients…to improve functional capacity, alleviate or lesson activity-related symptoms, reduce unwarranted invalidism, an enable the cardiac patient to return to a useful and personally satisfying role in society”(34).

Cardiac rehabilitation was generally accepted as being made up of sequential phases: Phase 1 focused on inpatient mobilization and introductory information; Phase 2 was an outpatient hospital-based program that was run in groups attending for approximately 6–12 weeks; and Phase 3 was known as a maintenance phase of 4–6 months duration when patients continued their exercise and risk factor modification routine while returning to their regular life and work (35). Each of these phases also included multidisciplinary education component that provided information about risk factors such as smoking cessation, healthy diet, medication adherence and psychosocial support (36). Programs varied slightly in terms of session frequency per week and duration, likely based on funding availability, given rehabilitation has not been funded in the same direct manner as acute cardiac care (21). In the United States, and many other countries, private health insurance funding systems have facilitated this model where most companies provide coverage for a program of several sessions per week for 8–12 weeks (rather than life-long prevention) (21).

Numerous systematic reviews have since found these exercise-based cardiac rehabilitation is beneficial for those who attend (37–39). These benefits for people with CHD include reduced risk of MI, a modest reduction in all-cause mortality, and a considerable reduction in all-cause hospital admissions along with associated healthcare costs and improved quality of life up to 12 months (37–39). However, despite international (40–42) guidelines now universally recommending cardiac rehabilitation and secondary prevention, rates of referral, access to programs and adherence to recommendations remained problematic (43, 44). Use of evidence-based medications and lifestyle change typically started to regress within the first 6 months and was rarely sustained (45, 46). Research has since consistently found only 30–50% of those eligible are referred, around 10% of those eligible actually attend structured programs and less than 5% of those initially eligible complete a full program of traditional cardiac rehabilitation (43, 44, 47). Reasons for these suboptimal attendance and completion figures are widely reported and include issues with transport, lack of flexibility and lack of perceived need balanced with work and social commitments of patients (47, 48). Further, certain groups are less likely to attend including women, those from culturally/linguistically diverse or low socioeconomic backgrounds (49). Further, if one takes a health systems view, the financial burden and practical requirements of providing a traditional program to all who are potentially eligible remains a formidable challenge (6).

## Changing Lifestyle, Medical, and Surgical Management: 2000s

In more recent years, lifestyle factors including cigarette smoking, poor diet, inactivity and sedentary behavior have become widely accepted as contributing to increased likelihood of events (50, 51). Emergence of technology and fast food availability have influenced societal behaviors, resulting in increasing sedentary behavior and poor diet (52). Further, the importance of psychosocial factors gained attention, with many programs expanding to include psychological support and stress management (21).

During the 2000s, cardiac rehabilitation programs tended to continue as they had been in the decades prior. Importantly, a global study published in 2019 sought to gather data about all phase 2 cardiac rehabilitation programs offered worldwide (21). Data was collected by an online survey shared by local leaders and stakeholder organizations. Results found that the majority (83%) offered exercise training, but few programs reported offering an alternative model: 12% of programs offered a home-based service, and/or 10% offered a community-based (21). Sessions included a mean of 9 patients (i.e., most being group-based with a mean of 5 patients per staff member). Further, the majority (83%) offered exercise training but only 26% of programs reported offering an alternative model (21). These findings are similar to those of a 2009 Australian policy statement that found 72% of programs follow the traditional cardiac rehabilitation model based on approximately 2 months of supervised group exercise and education (53). This lack of flexibility has been identified as a barrier to participation in cardiac rehabilitation (54). Research exploring barriers and enablers to participation and completion was expanding at this time, with increasing recognition that suboptimal proportions of eligible patients were attending (48). Achieving health systems reform to address this growing gap remains a key challenge for the healthcare community.

## The Societal Transformation and the Digital Era: 2010s

Globalization has resulted in economic development raising many countries out of poverty as well as enormous interdependence of the world’s cultures and populations (55). Of course this includes major impacts on health at individual, population and systems levels. For CVD and cardiac rehabilitation, the associated challenges include a greater need to manage equity and diversity both within and between countries. For example, individuals who do not speak the language of the country in which they live, those who live in rural and remote geographical areas those with socioeconomic disadvantage and women remain under-represented in cardiac rehabilitation. Between countries we also see enormous disparity; up to 90% of the worldwide CVD burden is carried by low- and middle-income countries (LMICs), while these countries often have very large populations coupled with a lack of resources (56). Ultimately, it is not feasible to offer traditional, group-based and in-person cardiac rehabilitation at scale to all people who are eligible. As such, inequity and poor reach of cardiac rehabilitation has become a major challenge for clinicians and policy-makers.

The so-called Digital Era in the 21st century has elicited enormous transformation in the way people communicate, behave and interact on all levels. As of January 2021, it is estimated that around 60% of the world’s population have internet access and 80% own a smartphone (57, 58). This has subsequently transformed health management with increased use of electronic devices to support health, now often referred to as digital health or eHealth. This technology affords new strategies for communication with patients. Examples in the literature where technology has supported patients with CVD include telephone coaching (59, 60), text message programs (61, 62), interactive online programs (63), smartphone apps (64), and the use of sensors and personal trackers to automatically monitor behavior (65). Digital health strategies also include electronic prescribing of medications, remote monitoring *via* Bluetooth devices and use of artificial intelligence linked to implantable devices to enable remote feedback and support in real-time (65). Such strategies can support tobacco cessation, diet, physical activity, mental health etc. However, despite promising developments from a technological perspective, there remains a lack of scientific evidence for effectiveness of some approaches, thus this is an increasingly active area of research.

In the early 2020s, the global acute respiratory syndrome (COVID-19) pandemic had a major impact on cardiac rehabilitation delivery around the world. Human transmission of infection with the novel coronavirus was first detected in late 2019 and rapidly spread. At the time of writing, there have been approximately 5.2 million deaths globally from the pandemic (66). COVID-19 has been responsible for enormous pressure on healthcare services and systems (67). In an effort to curb spread of the virus, hundreds of countries have enforced full or partial lockdown of their citizens which has of course impacted the lives and wellbeing of billions of people across the world (67). For patients with established CHD, the pandemic has resulted in enormous changes in access to the health care system such as reduced in-person medical appointments and closed cardiac rehabilitation services (68, 69). For cardiac rehabilitation, survey data suggest that approximately 4,400 programs (estimated 75% of programs around the world) ceased or were temporarily stopped due to COVID-19 (70). This has necessitated a dramatic shift from in-person models to home-based programs necessitating more widespread implementation of virtual and digital models of care (70). Many are now speculating about the future of cardiac rehabilitation and what format it should take, with calls from the International Council of Cardiovascular Prevention and Rehabilitation for ongoing availability of unsupervised delivery formats with associated reimbursement advocacy.

## Learnings From History to Inform Contemporary Recommendations

Since cardiac rehabilitation programs emerged, there have been enormous changes to both the medical and surgical care of patients with CVD coupled with transformation of societies and technology. Very few patients now need a period of “rehabilitation,” but rather life-long multifaceted prevention is needed to reduce the CVD burden. At the same time, even when offered, only the minority of eligible patients attend (traditional) cardiac rehabilitation programs and to meet current and projected expanding need within financial limits, contemporary models of cardiac rehabilitation are being modified so as to better align with other treatments, changing societies and technological advancements. Below, we suggest key recommendations that emerge with consideration of the history of cardiac rehabilitation as preventive cardiology moves forward into the new millennium:

1. Implementation of lifelong preventive strategies, rather than time-limited programs, would optimize continuous management and care for patients.
2. Building flexibility into cardiac rehabilitation delivery models to improve program reach and equity through for example, home-based programs, cultural and language tailoring, ensuring inclusivity with regard to diversity, cognitive impairment, geographical access etc. (54).
3. Systematic incorporation of cardiac rehabilitation into hospital performance measures, with digital integration such as automatic referral and standardized benchmarking (71).
4. Ensuring programs are focused on comprehensive risk factor management (not only exercise-based) based on individual patient need to optimize personalization of care across all relevant risk factors including psychosocial issues to optimize potential benefit of preventing new events (72).
5. Scientific evaluation of evidence for and implementation (where effective) of digital health interventions to support secondary prevention. Such strategies include communication *via* the telephone and internet which are now widely available, as well as use of mobile applications (apps), tracking sensors, text messaging and so on. These strategies have been evolving but have accelerated in availability as a result of the COVID-19 pandemic. However, robust trial/registry research is needed to continue to ensure effectiveness and usefulness for patients.

6. Focus on implementation of approaches that are tailored to the needs of LMICs where the CHD burden is greatest to improve access to and engagement with effective secondary prevention. Widespread availability of mobile technology offers a promising pathway to achieving this implementation although evidence-based strategies are needed (73).
7. Universal definition and classification of preventive “rehabilitation”; including cardiology, nursing, allied health, primary care, consumers, policy-makers is needed to demonstrate leadership and champion access and implementation of evidence-based care.
8. Advocacy for suitable reimbursement and funding of flexible models of cardiac rehabilitation.
9. Although controversial and potentially challenging, organizations and leading stakeholder groups could consider revisiting the term “rehabilitation” and revising to a more inclusive term such as “secondary prevention” or “preventive cardiology.” While this particular term was relevant in the 1970s, it may not be reflective of the full potential of secondary prevention programs in the 21st century.

## Conclusion

During the last 75 years there has been a reversal of inpatient and post-discharge care and treatment guidelines for patients with CHD. This historical overview highlights how modern-day cardiac rehabilitation was born over 50 years ago at a time when *bed rest and physical inactivity were commonplace*. Despite undergoing some reform, this traditional model is still followed by the majority of programs around the world which in itself is a major barrier to change. This is despite major changes in medical management and surgical approaches to CHD coupled with different sociocultural norms and technological development globally. Understanding this history enables consideration of opportunities for reform that include greater flexibility, the need for life-long prevention and the potential value of digital health in improving reach and sustainability of programs.

## Author Contributions

JR conceived and draft the manuscript. RG, AO’N, SG, and TB provided the rehabilitation expertise. AB, GJ, and DB gave policy, public health and cardiology input. All authors reviewed multiple versions and the manuscript and approval the final version.

## Conflict of Interest

The authors declare that the research was conducted in the absence of any commercial or financial relationships that could be construed as a potential conflict of interest.

## Publisher’s Note

All claims expressed in this article are solely those of the authors and do not necessarily represent those of their affiliated organizations, or those of the publisher, the editors and the reviewers. Any product that may be evaluated in this article, or claim that may be made by its manufacturer, is not guaranteed or endorsed by the publisher.
